# Leadership, Innovative Behavior and the Case of Innovative Climate—When the Mediator Becomes the Mediated

**DOI:** 10.3390/bs13010040

**Published:** 2023-01-02

**Authors:** Milan S Lecic, Bojana Milic, Ruzica Visnjic, Jelena Culibrk

**Affiliations:** Faculty of Technical Sciences, University of Novi Sad, 21000 Novi Sad, Serbia

**Keywords:** leadership, LMX, innovative climate, innovative behavior, internal trust, organizational commitment

## Abstract

This study investigates how leadership, more precisely leader–member exchange (LMX), affects innovative behavior through an innovative climate as well as, indirectly, through organizational commitment and internal trust. A total of 1114 samples were collected from employees working in firms in Slovenia and Serbia. The quantitative data and the proposed model were analyzed with the partial least squares—SEM technique. The results indicate that an innovative climate is a mediator in the relationship between leadership and innovative behavior, and this relationship is also further mediated by internal trust and organizational commitment. These results reveal serial mediation or the complex role of an innovative climate in the process of influencing innovative work behavior. Implications for theory and recommendations for practice are discussed.

## 1. Introduction

The recent COVID-19 pandemic, growing uncertainty in the world, and possibility of a recession are influencing both developed and developing countries, presenting challenges for organizations that are struggling to retain their operations and survive during these challenging times. In order to adapt, these organizations must rely on sound leadership and innovative employees to quickly develop and implement innovative strategies. Failure to innovate can expose organizations to potential risk and reduce their ability to survive or to gain a competitive advantage [1]. This study aims to investigate how leadership, more precisely leader–member exchange (LMX), affects innovative behavior through an innovative climate. In previous studies, an innovative climate is sometimes identified as a mediator of said relationship. However, those studies failed to investigate the possibility of an innovative climate also being mediated by other organizational factors. In this study we propose the so-called “mediation of the mediator” and aim to investigate the indirect effects through internal trust and organizational commitment. This study presents a model that illustrates said relationships and seeks to explain the complex role of an innovative climate in the process of influencing innovative work behavior.

## 2. Literature Review and Hypotheses Development

### 2.1. Leadership and Innovative Climate

An effective leader is considered to be one who is able to successfully prepare a company for upcoming challenges. They anticipate change and the future with their vision. An effective leader is able to foster a sense of personal commitment and to transform individuals into important prospects [2]. Leadership should be viewed as a process that allows employees to embrace the vision, while facilitating individual and collective learning and the achievement of organizational goals [3]. The leader–member exchange (LMX) theory focuses on the interaction between leaders and followers [4]. The leadership-making model proposed by Graen and Uhl-Bien [5] highlights the importance of a leader’s high-quality exchanges with their followers. Those exchanges allow leaders to give their followers something other than working just for self-gain and to offer new responsibilities and roles. In exchange, followers feel loyalty and trust, are willing to do more, and seek innovative ways to work harder than expected. While focusing on the exchanges with their followers, leaders also influence the attitudes, emotions, and behaviors that make up the organizational climate [6]. Organizational climate can be viewed as the organizational experience [7] or as a collective perception of the organization [8] that can be expressed through trust, openness, commitment, motivation, and a willingness to take risks [6]. According to Isaksen and Ekvall [9], the greatest challenge for modern leaders is to foster an organizational climate that encourages and promotes creative thinking and innovation. Only in that case will organizations be able to acquire and retain their competitive advantage, grow, and increase economic development [10]. An innovative climate is an internal environment that supports innovation and change, which is crucial for innovative organizations [11], so that employees can share and build on ideas with each other [9]. An innovative climate is, therefore, a set of the employees’ perceptions of the organization’s work environment that encourages creativity and the use of creative approaches regarding the allocation of resources, the execution of work activities, and the creation of new products and services [12].

The impact of leadership on organizational climate has long been studied [13,14], but there is still great interest in researching the impact of leadership on an innovative climate. There is evidence that a leader’s behavior can influence an innovative climate [15,16,17]. Scott and Bruce [12] found that LMX can predict an innovative climate: a higher level of leader–member interaction indicates a greater perceived climate for innovation. These results were confirmed in a study conducted by Kazama and colleagues [18].

### 2.2. Innovative Climate and Innovative Behavior

Employees’ creativity and innovation are the driving forces for change that allow organizations to build and secure their future [19,20,21,22], so it is crucial to understand the creation and the dynamics of an organizational climate that will encourage the employees’ innovative behavior [23,24,25]. Innovative work behavior includes a broad set of behaviors related to generating and promoting ideas, creating support for them, and helping their realization and implementation [26,27]. An innovative climate encourages employees to be committed to the organization and to work toward achieving organizational goals. This is because as soon as employees perceive a positive climate that is conducive to innovation, they tend to actively participate in innovations instead of remaining passive [28,29]. It has been found that an innovative climate not only supports employees’ innovative behavior [30,31] but also supports their motives and attitudes toward innovation [32]. Recent research conducted by Odoardi et al. suggests that employees will be more willing to accept innovation goals or to demonstrate innovative behavior if they perceive the work environment as a place where ideas are truly appreciated and where innovative and creative endeavors are valued and nurtured [33]. Previous research suggests that certain dimensions of an innovative climate, such as autonomy and freedom, along with specialized knowledge, have a positive effect on innovative behavior [34]. When employees become aware that freedom exists in their work environment, they are more willing to take initiative, promote their own ideas, and propose new processes, while increasing their innovativeness [15,35].

### 2.3. Mediating Role of Innovative Climate

Mumford and Gustafson [17] and Jung [36] showed that leadership is one of the most important, if not the most important, influencing factors on employee creativity and innovative performance. De Jong [37], who conducted in-depth research with leaders in knowledge-intensive businesses, found that an innovative climate is a precursor to innovative work behavior. West [38] had a similar attitude in their research and stated that creative and innovative behavior is a product of the combination of personal qualities and factors in the work environment. According to Moghimi and Subramaniam [39], the components of an innovative climate that significantly predict the innovative behavior of employees are the resources that encourage creativity and innovation and the clarity of a mission that refers to the awareness of goals and expectations regarding creative performance, as well as support from the leader. Describing what leaders can do to manage creativity and innovation, Catmull [40] pointed out that leaders need to build an environment that fosters relationships of trust and respect in order to release creativity in the organization. Leaders who know how to positively influence an innovative climate and innovative behavior that creates innovation, create the potential for innovation resulting in increased performance [41]. Given that leadership and an innovative climate influence innovative behavior, an innovative climate is identified as an intervening variable. Few studies have examined how an innovative climate mediates the relationship between leadership and innovative outcomes. Ekvall and Ryhammer [42,43] found evidence for the moderating role of an organizational climate when investigating leadership styles and organizational outcomes at a Swedish university. Jung, Chow, and Wu [44] studied the effects of transformational leadership, empowerment, and innovation support. They found that transformational leadership was significantly and positively related to organizational innovation and innovation support and also concluded that the leadership styles of top managers can significantly influence the creativity and innovative capabilities of an organization. They believe that the main way to gain this benefit is to create an organizational climate that encourages employees and supports innovation. An innovative climate is important for achieving organizational results and can serve as a means for leaders to positively influence organizational performance. Company leaders should create an organizational climate that inspires and supports innovative behaviors to facilitate the impact of their leadership style on company performance [45].

### 2.4. Mediating Role of Internal Trust

Trust is defined as “the willingness of a party to be vulnerable to the actions of another party based on the expectation that the other will perform a particular action important to the trustor, irrespective of the ability to monitor or control that other party” [46]. In order for trust to exist, there must be an expectation of reliability and an intention in the trustee’s behavior to perform as expected [47]. Trust involves a degree of uncertainty and involves belief in their partner’s abilities and benevolence. Therefore, the emphasis is on the readiness of the trustor to show trust, not on the trustworthiness of the trustee. Internal trust represents the climate of trust in an organization. It is defined as “positive expectations individuals have about the intent and behaviors of multiple organizational members, based on organizational roles, relationships, experiences and interdependencies”. Organizations will be more innovative, adaptive, and successful if they manage to obtain a great deal of internal trust [48]. Most existing studies suggest that the process of building internal trust lies in the hands of the leader. Baer and colleagues [49] suggested that an employee’s feelings of trust develop in response to an actual trusting behavior by a supervisor, such as delegating crucial tasks or disclosing sensitive information. Andersen [50] revealed a connection between the actions of superiors and the trust of their subordinates. Superiors gain the trust of their subordinates through their activities, so the level of trust differs across different levels of a hierarchy. A leader’s efforts to build trust are also identified as one of the key mechanisms to increase organizational effectiveness [51]. Research conducted by Pučetaite, Noveskaite, and Markunaite [52] confirms that the leadership’s attitude toward employees can improve trust in private organizations. A correlation between leadership and internal trust was found by several studies [53,54].

On the other hand, internal trust can be found to facilitate the exchange of ideas and to reduce their complexity, but it also lowers the fear of failure and criticism when implementing new ideas [55]. Krot and Lewicka [56] found a positive relationship between internal trust and the creation of an innovative climate. A strong relationship is found between internal trust and innovation, and there is a strong suggestion that trust should be treated as the foundation of innovation [57]. Curseu and Schruijer [58] found that a low level of trust can make people feel attacked when disclosing information; this makes it less likely that the release of knowledge and the creation of an innovative climate in which innovation can be realized would follow.

### 2.5. Mediating Role of Organizational Commitment

Organizational commitment is seen as a form of psychological connection between employees and the organization. Porter [59] defined organizational commitment as the “strength of an individual’s identification with and involvement in a particular organization”, consisting of three factors: (1) a strong belief in and acceptance of the organization’s goals and values, (2) a willingness to exert considerable effort on behalf of the organization, and (3) a definite desire to maintain organizational membership. Sheldon [60] proposed that commitment is a positive attitude toward the organization and an intention to work toward achieving its goals. A significant number of research studies showed that leadership behavior has a very strong and positive effect on organizational commitment [61,62,63,64,65]. If leaders exhibit supportive, directive, and participative leadership behaviors, employees have high levels of commitment and involvement with their organization [66]. Similarly, Lok and Crawford [67] found that leadership style positively affects the level of employee commitment. Also, Stum [68] implied that leadership has a significant correlation or relationship with employee commitment and suggested a positive direct relationship between leadership behavior and employee commitment. The results of a study conducted by Nangoli et al. [69] showed that perceived leadership integrity, as a leadership attribute, provides a basis for the creation of organizational commitment, i.e., that perceived leadership integrity has a positive impact on employee commitment. This finding is consistent with earlier studies [70,71].

Research conducted by Lin [72] confirmed a significant positive correlation between an innovative climate and organizational commitment. They proposed that improving a company’s innovative climate and organizational commitment can effectively positively influence the performance of the company. An innovative climate can affect commitment through activities that increase the autonomy, competence, and relatedness of employees [73]. From the perspective of the self-determination theory, an innovative climate is associated with self-determination, when employees experience autonomy and competence and try new ideas [74], i.e., employees have a greater affective commitment when there is a better climate for innovation. When a leader supports new ideas, shares ideas, encourages innovation, and provides sufficient resources and time, their employees tend to show greater commitment [75,76]. Thus, a positive organizational commitment can increase an organization’s innovative climate.

Based on the previous literature review, the following hypothesis are developed:

H1 Leader–member exchange (LMX) positively affects an innovative climate (IC);
H2 An innovative climate (IC) positively affects innovative behavior (IB);
H3 Innovative behavior (IB) is positively affected by leader–member exchange (LMX) through the mediating role of an innovative climate (IC);
H4 Leader–member exchange (LMX) positively affects internal trust (IT);
H4a An innovative climate (IC) is positively affected by Leader–member exchange (LMX) through the mediating role of internal trust (IT);
H5 Leader–member exchange (LMX) positively affects organizational commitment (OC);
H5a An innovative climate (IC) is indirectly affected by leader–member exchange (LMX) through the mediating role of organizational commitment (OC).

**Figure 1:**
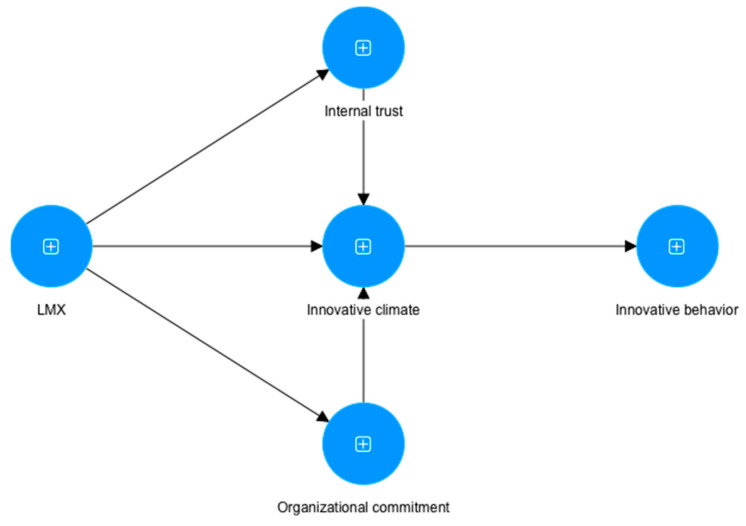
The conceptual model.

The conceptual model based on these hypotheses is presented in Figure 1.

## 3. Materials and Methods

### 3.1. Measurement

In the theoretical model, five constructs were defined. The questionnaire used in this study consisted of 10 questions about the respondent’s demographic, while 41 items were used to measure the constructs. The measures of constructs used in this study were adapted from previous research identified in an extensive literature review. Leader–member exchange was measured with the LMX-7 scale [77]. The Organizational Commitment Questionnaire—OCQ was used in order to measure organizational commitment [78]. This consisted of 15 items, 6 of which were negatively formulated. Internal trust was measured with four items adapted from Huff and Kelley’s [79] previous study. Innovative climate was measured with five items that evaluate the innovative dimension of the psychological climate [7]. Innovative work behavior was measured with the 10-item Innovative Work Behavior Scale—IWBS [80]. Respondents evaluated the items on a 5-point Likert scale, with response options ranging from 1 for “completely disagree” to 5 for “completely agree”. For the purpose of this study, the items were translated from English to Serbian and Slovenian by the double translation method [81]. Firstly, the questionnaire was translated from the English language by licensed translators. Secondly, by back-translation, the questionnaires were translated back to the English language by two people who are management experts and fluent in English, Serbian, and Slovenian. The double translation method showed that the translation process did not change the essence of the items. The questionnaires were also separately validated for internal consistency, validity, and reliability. Confirmatory factor analysis showed that all measures of internal consistency, validity, and reliability showed very similar values for both the Serbian and the Slovenian versions of the questionnaire. Based on these results, it was concluded that there is justification for the use of adapted scales for Serbian and Slovenian, so the following analyses used a summarized sample from Serbia and Slovenia.

### 3.2. Data Collection and Sample Characteristics

The quantitative data were obtained from May to October 2021. The sample size was projected based on Hair’s [82] suggestions for PLS-SEM analysis. In order to achieve the statistical power of the test of 80% and to obtain the R^2^ value of 0.25 at the statistical significance level of 95%, for a total of 5 variables in the model, it was necessary to provide a minimum of 45 observations [83]. Leading companies from different fields operating in Serbia and Slovenia were contacted via email. The questionnaire was sent to a company’s PR or HR representatives when they responded positively to the invitation for participation in this study. The research was approved by the top management of the involved organizations. A total of 69 companies were contacted (38 from Serbia and 31 from Slovenia), while a total of 32 companies responded positively (18 from Serbia and 14 from Slovenia). Since anonymity of respondents was requested, and companies distributed questionnaires through company email, the exact number of distributed questionnaires is unknown. The respondents accessed the questionnaire through the Google Form platform. The final sample consisted of 1114 respondents from Serbia (54%) and Slovenia (46%), which exceeded the identified minimum sample size. Sample demographics are presented in Table 1.

## 4. Results

Partial least squares structural equation modeling (PLS-SEM) is able to investigate multiple relationships between variables at the same time, while focusing on prediction [84]. It is carried out through the development and analysis of measurement and structural models, which are used to assess the fit of the model; through a series of analyses, the model examines the consistency between the assumed theoretical model and the model obtained by measurement in the population [85]. PLS-SEM was conducted using SmartPLS 4 software [86]. In the first step, the measurement model was analyzed, while, in the second step, the structural model was tested.

### 4.1. Measurement Model

In structural equation modeling, the relationships in the model are viewed at two levels. At the first level, we observe the relationship between a construct and its indicators. These relationships make up the measurement model [85]. Reflective measurement models are assessed for convergent validity, internal consistency reliability, and discriminant validity [83]. Convergent validity was assessed by examining the indicator’s other loadings and AVE. Hair [85] suggested that the minimum value for outer loading should be 0.70. Indicators with outer loadings ranging from 0.4 to 0.70 should be excluded from the measurement model if they are found to affect the overall composite reliability. As presented in Table 2, items with an outer loading lower than 0.70 (highlighted in bold) were excluded from the measurement model. The next indicator used to assess convergent validity is AVE. According to Hair [85], a value greater than 0.5 should be taken as the threshold value, because it indicates that the construct explains at least 50% of the variance in reflective indicators. Since all constructs met this criterion, it was concluded that convergent validity has been established (Table 2). Internal consistency reliability was evaluated through Cronbach’s alpha and CR values. The general rule for Cronbach’s alpha is that values higher than 0.70 are desirable, values above 0.80 are better, and values above 0.90 are considered the best [87]. On the other hand, Hair and colleagues suggested that the threshold values for CR should range from 0.70 to 0.90. As shown in Table 2, the minimum value for Cronbach’s alpha was 0.870, while the minimum value for CR was 0.658, indicating an acceptable level of internal consistency.

Finally, discriminant validity, which assesses validity in relation to other constructs in the model, was examined through the Fornell–Larcker criterion and the HTMT criterion. The Fornell–Larcker criterion compares the correlation between constructs with the values of the square roots of the construct’s AVE. As shown in Table 3, all values in the columns are lower than the squared roots of AVE in the corresponding column. These results indicate that the Fornell–Larcker criterion is fully met.

Using the HTMT criterion, we assume the correlation coefficients were measured without error. The upper threshold value for HTMT is 0.85 [88]. In Table 4, it can be seen that the values of the HTMT criterion do not exceed the recommended value of 0.85. We also tested the confidence intervals for HTMT with bootstrap analysis, and these results showed that value 1 was not found in the confidence intervals. These results confirm the discriminant validity of constructs.

### 4.2. Structural Model

**Figure 2:**
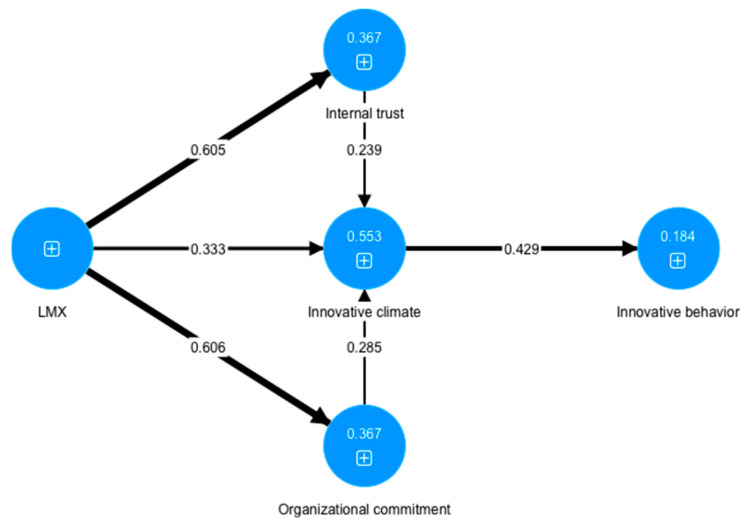
The structural model.

A structural model represents the relationships between the constructs in the model. The links between the constructs in the model correspond to the hypotheses based on the theoretical model (Figure 2). The structural model was tested with the PLS algorithm and bootstrapping. The structural model was first tested for collinearity by examining the VIF values, and the results showed no collinearity issues in the structural model, since all the VIF values (VIF (LMX->OC) = 2077; VIF (LMX->IT) = 2077; VIF (LMX->IC) = 1777) were below the conservative threshold of 3, as suggested by Hair and colleagues [83].

The hypotheses were tested with path analysis (Table 5). All the direct and indirect effects examined in the structural model were statistically significant. If we look at the strength of the relationships between the constructs, the strongest effect (β > 0.6) was obtained for paths LMX-> OC (H5) and LMX-> IT (H4). An effect of somewhat weaker intensity (β = 0.3–0.6) was obtained for paths IC -> IB (H2) and LMX-> IC (H1). The effect of the weakest intensity (β < 0.3) was obtained for paths OC-> IC (H2) and OC-> IC (H3) as well as for the mediation paths (H4a and H4b). Mediation analysis observed the direct effect LMX-> IC and indirect effects through OC and IT. Since both the direct and indirect effects were statistically significant, and the path coefficients had the same sign, these mediations were categorized as complementary mediations, as suggested by Hair and colleagues [84] and Zhao and colleagues (2010). This meant that in the relationship between LMX and IC, the constructs OC and IT act as complementary mediators that strengthen the existing relationship. Based on these results, we concluded that all assumed hypotheses (H1–H5) were supported.

The coefficient of determination R^2^ measures how well a structural model predicts an outcome [83]. The results show that the highest value of R^2^ was obtained for IC (R^2^ = 0.553). Equal values of the R^2^ coefficient were obtained for the constructs IC and IT (R^2^ = 0.367). The weakest R^2^ was identified for IB (R^2^ = 0.184). To assess the predictive validity of the structural model, the Q^2^ value is used, which is calculated using the PLSpredict algorithm. According to Hair [83], Q^2^ values greater than zero confirm the predictive validity of the structural model. All the values of Q^2^ for the latent variables were greater than 0, which indicated the good predictive power of the structural model. The highest values of predictability were obtained for the construct IC (Q^2^ = 0.422), followed by the OC and IT (Q^2^ = 0.365). The IB showed the lowest predictive power (Q^2^ = 0.126). By using the importance–performance maps, we can determine which predictor is the most significant in order to have an impact on a certain latent variable. By analyzing the importance–performance map for IC, it was determined that L shows the highest values for both importance and performance for the latent variable IC. Analysis of the importance–performance map for IB showed that IC has the highest values for both importance and performance for the latent variable IB.

## 5. Discussion

This study investigates how leadership directly affects innovative behavior through an innovative climate and indirectly affects innovative behavior through organizational commitment and internal trust. The findings indicate that an innovative climate is a mediator in the relationship between LMX and innovative behavior but that this relationship is further mediated by internal trust and organizational commitment (Table 5). These results reveal the serial mediation or the complex nature of an innovative climate in the process of influencing innovative work behavior.

### 5.1. Theoretical Implications

This study focuses on the role of an innovative climate in the process of influencing employees’ innovative behavior. Previous results indicated that leader behavior can influence innovative climate [12]. Studies that investigated the impact of leader–member exchange on innovative work behavior with the mediating role of an innovative climate identified the mediating effect of an innovative climate, ultimately suggesting that the importance of an innovative climate may have been overstated in previous research [12]. The presented results reveal the complex role of an innovative climate in the process of influencing employees’ innovative work behavior, suggesting that an innovative climate can further be influenced by other organizational factors, such as internal trust and organizational commitment, since more than 55% of an innovative climate’s variance is explained by the proposed model. These results indicate that an innovative climate should not be viewed as a situational variable that is independent of other organizational factors. As the results of previous studies suggested, leaders play an important role in creating and supporting both internal trust [52,89,90] and organizational commitment [70,91,92]. Internal trust and organizational commitment are identified as complementary mediators that further strengthen the relationship between leadership and an innovative climate and innovative behavior. It has been previously found that trust in managers is highly dependent on their actions, suggesting that trust is the key concept of contemporary leadership [50]. Leaders who establish loyal and caring relationships can improve trust in organizations [52], while creating an environment that promotes creativity and supports new ideas. The most recent research showed that organizational commitment is an important indicator of innovative behavior as well as job performance [93]; although this research included an innovative climate, it did not investigate its mediating role. Despite the fact that an innovative climate has a strong and positive relationship with innovative behavior (β = 0.429), our results show that the tested model explained up to 20% of total variance of innovative behavior. Bain et al. suggested that different intensities of the relationship between an innovative climate and innovative behavior can be found when comparing (1) team and individual level, where a weaker relationship is found on individual level ajd (2) duration of the project, where a stronger relationship is found for long-term projects [94]. Scott and Bruce suggested that future researchers should focus on finding the direct effects that influence innovative behavior [12], and the results of this study support those assumptions. More recent research investigated other mediators in the relationship between LMX and innovative behavior, such as perceived organizational support [95], employee voice [96], and proactivity [97]. Even though the main focus of our proposed model was to further explain employees’ innovative behavior, the results indicate that it performed much better at explaining an innovative climate. Our results provide clear empirical support for the importance of an innovative climate but also reveal its susceptible nature to the influence of other organizational factors, which is the most important implication for theoretical development. While searching for factors that could directly influence employees’ innovative behavior, researchers should not consider these factors individually; rather, they need to be investigated in the wider context of an organization. This implies designing complex theoretical models that will include leadership, a creative climate, and innovative behavior in relation to organizational factors such as motivation, communication, organizational support, etc. Simultaneous analysis of complex models can provide more detailed insight into the nature of innovative behavior in organizations.

### 5.2. Practical Implications

The results of this study imply that positive leader–member exchange and an innovative climate are not sufficient for innovative behavior. Organizations and leaders should strive to be effective in creating an institutional framework and supportive an innovative climate in which creativity and innovation will be accepted as basic norms in the midst of technological and other changes [19]. The results suggest that leaders can influence employees’ innovative behavior indirectly, by establishing a climate that encourages them and supports innovation. This is consistent with earlier results showing that if employees perceived their leader to be more effective, they observed a better climate for innovation [98]. The results of the current study suggest that, after leaders make an effort to improve internal trust and organizational commitment, they should continue to leverage the benefits of these organizational factors. This way, leaders can encourage an open exchange of ideas by their committed employees, who are willing to go further without the fear of criticism or suppression of their ideas.

## 6. Limitations and Future Directions

There are several limitations that should be addressed. Our research was conducted in two countries; this should be expanded to further investigate different contexts. The study used cross-sectional data that have limitations when assessing causality. Also, the constructs were investigated with self-assessment instruments that lack objectivity. These methodological problems could be overcome by combining experimental research to investigate innovative behavior, through a longitudinal study design to investigate causality. It would be recommended to investigate how different leadership styles influence innovative behavior and how these styles may interact with LMX. In addition, it would be very interesting to observe the results for employees in managerial and non-managerial positions. Future studies should also include other constructs that may have a direct effect on innovative behavior and explore other possible mediators and moderators that were not included in this study.

## 7. Conclusions

This study proposes recommendations for company leaders in both developed and underdeveloped countries. During times of crisis, innovation becomes even more important for companies because their survival depends on it. Leaders should build an innovative environment that encourages out-of-the-box thinking, generating new knowledge and ideas and promoting and implementing new and improved ideas and business processes. Leaders also need to stimulate internal trust with open communication about the real state of affairs, by trusting their employees with delicate and important tasks in such challenging times. Committed employees will not only remain with the organization when a crisis occurs, but will try with all their might to find new solutions to help overcome the situation. Based on the presented results, we believe that this research has contributed to the identification of gaps in the literature and the improvement of theoretical and empirical implications.

## Figures and Tables

**Table 1:** Sample demographics.

Variable	Category	N	%
Country	Serbia	597	53.6%
	Slovenia	517	46.4%
Gender	Female	579	52%
	Male	535	48%
	Other	0	0%
Age	<25 yrs.	29	2.6%
	25–34 yrs.	244	21.9%
	35–44 yrs.	318	28.5%
	45–54 yrs.	302	27.1%
	>55 yrs.	221	19.8%
Level of education	Elementary school	36	3.2%
	High school	381	34.2%
	College	231	20.7%
	University (B.A. and M.Sc.)	382	34.3%
	University (Ph.D.)	84	7.5%
Total work experience	<2 yrs.	201	18%
	2–4 yrs.	203	18.2%
	5–9 yrs.	204	18.3%
	10–14 yrs.	169	15.2%
	15–19 yrs.	110	9.9%
	>20 yrs.	227	20.4%
Work experience in current company	<2 yrs.	34	3.1%
	2–4 yrs.	98	8.8%
	5–9 yrs.	134	12%
	10–14 yrs.	145	13%
	15–19 yrs.	141	12.7%
	>20 yrs.	562	50.4%
Job position	Managerial	350	31.4%
	Non-managerial	764	68.6%
Company ownership	Public	413	37.1%
	Private	694	62.3%
	Other	7	0.6%
Type of company	Manufacturing	223	20%
	Service	727	65.3%
	Other	164	14.7%
Company operations	Local	700	62.8%
	International	414	37.2%
Size of company	<10 employees	217	19.5%
	10–49 employees	276	24.8%
	50–249 employees	275	24.7%
	>250 employees	346	31.1%

**Table 2:** Convergent validity and internal consistency reliability.

Constructs	Items	Outer Loadings	α	CR	AVE
Leader–member exchange	L_1	**0.638**	0.870	0.906	0.658
(LMX)	L_2	0.831			
	L_3	0.827			
	L_4	**0.687**			
	L_5	0.717			
	L_6	0.799			
	L_7	0.818			
Organizational commitment	OC_1	**0.638**	0.923	0.938	0.658
(OC)	OC_2	0.828			
	OC_3R	**0.378**			
	OC_4	**0.609**			
	OC_5	0.802			
	OC_6	0.864			
	OC_7R	**0.171**			
	OC_8	0.817			
	OC_9R	**0.568**			
	OC_10	0.791			
	OC_11R	**0.444**			
	OC_12R	**0.533**			
	OC_13	0.716			
	OC_14	0.800			
	OC_15R	**0.552**			
Internal trust (IT)	IT_1	0.881	0.910	0.936	0.787
	IT_2	0.903			
	IT_3	0.892			
	IT_4	0.871			
Innovative climate (IC)	IC_1	0.892	0.924	0.946	0.815
	IC_2	0.896			
	IC_3	0.882			
	IC_4	0.926			
	IC_5	**0.491**			
Innovative behavior (IB)	IB_1	**0.541**	0.924	0.942	0.669
	IB_2	**0.651**			
	IB_3	0.811			
	IB_4	0.770			
	IB_5	0.804			
	IB_6	0.828			
	IB_7	0.806			
	IB_8	0.815			
	IB_9	0.834			
	IB_10	0.842			

^1^ α—Cronbach’s α; ^2^ CR—composite reliability; ^3^ AVE—average variance extracted.

**Table 3:** Fornell–Larcker criterion.

Constructs	Mean	SD	LMX	OC	IT	IB
LMX	3.29	0.83	**0.811**			
OC	3.44	0.90	0.606	**0.828**		
IT	3.31	0.92	0.605	0.677	**0.887**	
IC	3.31	1.01	0.650	0.648	0.634	**0.903**
IB	3.32	0.83	0.369	0.472	0.383	0.429

Square root of AVE in bold on diagonal.

**Table 4:** Heterotrait–monotrait (HTMT) ratios.

Constructs	LMX	OC	IT	IC
OC	0.672			
IT	0.680	0.736		
IC	0.723	0.699	0.688	
IB	0.406	0.507	0.414	0.459

Square root of AVE in bold on diagonal.

**Table 5:** Path coefficients—direct and indirect effects.

Hypothesis	Relationship	Path Coefficient—β	*p*-Value	Decision
H1	LMX-> IC	0.333	0.000	Supported
H2	IC -> IB	0.429	0.000	Supported
H3	LMX-> IC-> IB	0.143	0.000	Supported
H4	LMX-> IT	0.605	0.000	Supported
H4a	LMX-> IT-> IC	0.172	0.000	Supported
H5	LMX-> OC	0.606	0.000	Supported
H5a	LMX-> OC-> IC	0.145	0.000	Supported

## Data Availability

The data presented in this study are available on request from the corresponding authors.

## References

[1] France C., Mott C., Wagner D. (2007). The innovation imperative: How leaders can build an innovation engine. Oliver Wyman J..

[2] Judge T.A., Bono J.E., Ilies R., Gerhardt M.W. (2002). Personality and leadership: A qualitative and quantitative review. J. Appl. Psychol..

[3] Yukl G.A. (2006). Leadership in Organizations.

[4] Northouse P.G. (2019). Introduction to Leadership: Concepts and Practice.

[5] Graen G., Uhl-Bien M. (1991). The Transformation of Professionals into Self-Managing and Partially Self-Designing Contributors: Toward a Theory of Leadership-Making. J. Manag. Syst..

[6] Ekvall G., Bass I.B.M., Drent P.J.D. (1987). The Climate Metaphor in Organisational Theory. Advances in Organizational Psychology: An International Review.

[7] Koys D.J., DeCotiis T.A. (1991). Inductive measures of psychological climate. Hum. Relat..

[8] Burton R.M., Lauridsen J., Obel B. (2004). The impact of organizational climate and strategic fit on firm performance. Hum. Resour. Manag..

[9] Isaksen S., Ekvall G. (2010). Managing for Innovation: The Two Faces of Tension in Creative Climates. Creat. Innov. Manag..

[10] DiLiello T.C., Houghton J.D. (2006). Maximizing organizational leadership capacity for the future: Toward a model of self-leadership, innovation and creativity. J. Manag. Psychol..

[11] Kissi J., Dainty A., Liu A. (2012). Examining middle managers’ influence on innovation in construction professional services firms: A tale of three innovations. Constr. Innov..

[12] Scott S.G., Bruce R.A. (1994). Determinants of innovative behavior: A path model of individual innovation in the workplace. Acad. Manag. J..

[13] Lewin K., Lippitt R., White R.K. (1939). Patterns of aggressive behavior in experimentally created “social climates”. J. Soc. Psychol..

[14] Litwin G.H., Stringer R.A., Harvard U. (1968). Motivation and Organizational Climate.

[15] Amabile T.M., Conti R., Coon H., Lazenby J., Herron M. (1996). Assessing the work environment for creativity. Acad. Manag. J..

[16] Mumford M.D., Scott G.M., Gaddis B., Strange J.M. (2002). Leading creative people: Orchestrating expertise and relationships. Leadersh. Q..

[17] Mumford M.D., Gustafson S.B. (1988). Creativity syndrome: Integration, application, and innovation. Psychol. Bull..

[18] Kazama S., Foster J., Hebl M., West M., Dawson J. (2002). Impacting climate for innovation: Can CEOs make a difference. Proceedings of the 17th Annual Conference of the Society for Industrial and Organizational Psychology.

[19] Ahmed P.K. (1998). Culture and climate for innovation. Eur. J. Innov. Manag..

[20] Gumusluoglu L., Ilsev A. (2009). Transformational leadership, creativity, and organizational innovation. J. Bus. Res..

[21] Martins E.C., Terblanche F. (2003). Building organisational culture that stimulates creativity and innovation. Eur. J. Innov. Manag..

[22] Pitta D.A. (2009). Creating a culture of innovation at Portugal Telecom. J. Prod. Brand Manag..

[23] Deshpandé R., Farley J.U. (2004). Organizational culture, market orientation, innovativeness, and firm performance: An international research odyssey. Int. J. Res. Mark..

[24] Nybakk E., Jenssen J.I. (2012). Innovation strategy, working climate, and financial performance in traditional manufacturing firms: An empirical analysis. Int. J. Innov. Manag..

[25] Patterson M., Warr P., West M. (2004). Organizational climate and company productivity: The role of employee affect and employee level. J. Occup. Organ. Psychol..

[26] Janssen O. (2000). Job demands, perceptions of effort-reward fairness and innovative work behaviour. J. Occup. Organ. Psychol..

[27] Scott S.G., Bruce R.A. (1998). Following the leader in R&D: The joint effect of subordinate problem-solving style and leader-member relations on innovative behavior. IEEE Trans. Eng. Manag..

[28] Luo Y., Cao Z., Lu Y., Zhang H., Wang Z. (2018). Relationship Between Extraversion and Employees? Innovative Behavior and Moderating Effect of Organizational Innovative Climate. Neuroquantology.

[29] Eckes A., Großmann N., Wilde M. (2018). Studies on the effects of structure in the context of autonomy-supportive or controlling teacher behavior on students’ intrinsic motivation. Learn. Individ. Differ..

[30] Liang Y., Zhihe Z. (2017). A mixed mechanism model of organizational innovation climate influencing the employee innovation behavior. Sci. Res. Manag..

[31] Wang H., Chang Y. (2017). The influence of organizational creative climate and work motivation on employee’s creative behavior. J. Manag. Sci..

[32] Zhu J., Li J., Wang X. (2017). How online community climate influences members’ innovation: An exploration from self-determination theory. Sci. Technol. Prog. Policy.

[33] Odoardi C., Battistelli A., Montani F. (2010). Can goal theories explain innovative work behaviour? The motivating power of innovation-related goals. BPA-Appl. Psychol. Bull..

[34] Krause D.E., Gebert D., Kearney E. (2007). Implementing process innovations: The benefits of combining delegative-participative with consultative-advisory leadership. J. Leadersh. Organ. Stud..

[35] Si S., Wei F. (2012). Transformational and transactional leaderships, empowerment climate, and innovation performance: A multilevel analysis in the Chinese context. Eur. J. Work Organ. Psychol..

[36] Jung D.I. (2001). Transformational and transactional leadership and their effects on creativity in groups. Creat. Res. J..

[37] Jong J.P.J. (2007). Individual Innovation: The Connection between Leadership and Employees’ Innovative Work Behavior.

[38] West M.A. (2002). Sparkling Fountains or Stagnant Ponds: An Integrative Model of Creativity and Innovation Implementation in Work Groups. Appl. Psychol..

[39] Moghimi S., Subramaniam I.D. (2013). Employees’ Creative Behavior: The Role of Organizational Climate in Malaysian SMEs. Int. J. Bus. Manag..

[40] Catmull E. (2008). How Pixar Fosters Collective Creativity.

[41] Shanker R., Bhanugopan R., van der Heijden B.I.J.M., Farrell M. (2017). Organizational climate for innovation and organizational performance: The mediating effect of innovative work behavior. J. Vocat. Behav..

[42] Ekvall G., Ryhammar L. (1998). Leadership style, social climate and organizational outcomes: A study of a Swedish University College. Creat. Innov. Manag..

[43] Ekvall G., Ryhammar L. (1999). The creative climate: Its determinants and effects at a Swedish university. Creat. Res. J..

[44] Jung D.I., Chow C., Wu A. (2003). The role of transformational leadership in enhancing organizational innovation: Hypotheses and some preliminary findings. Leadersh. Q..

[45] Puni A., Hilton S.K., Mohammed I., Korankye E.S. (2022). The mediating role of innovative climate on the relationship between transformational leadership and firm performance in developing countries: The case of Ghana. LODJ.

[46] Mayer, Davis J.H., Schoorman F.D. (1995). An Integrative Model Of Organizational Trust. AMR.

[47] Moorman C., Deshpandé R., Zaltman G. (1993). Factors Affecting Trust in Market Research Relationships. J. Mark..

[48] Shockley-Zalabak, Ellis K., Winograd G. (2000). Organizational trust: What it means, why it matters. Organ. Dev. J..

[49] Baer M.D., Dhensa-Kahlon R.K., Colquitt J.A., Rodell J.B., Outlaw R., Long D.M. (2015). Uneasy lies the head that bears the trust: The effects of feeling trusted on emotional exhaustion. Acad. Manag. J..

[50] Andersen J.A. (2005). Trust in managers: A study of why Swedish subordinates trust their managers. Bus. Ethics A Eur. Rev..

[51] Barney J.B., Hansen M.H. (1994). Trustworthiness as a source of competitive advantage. Strateg. Manag. J..

[52] Pučėtaitė R., Novelskaitė A., Markūnaitė L. (2015). The Mediating Role of Leadership Relationship in Building Organisational Trust on Ethical Culture of an Organisation. Econ. Sociol..

[53] Joseph E.E., Winston B.E. (2005). A correlation of servant leadership, leader trust, and organizational trust. Leadersh. Organ. Dev. J..

[54] Van Dierendonck D. (2011). Servant leadership: A review and synthesis. J. Manag..

[55] Luhmann N. (1979). Trust and Power.

[56] Krot K., Lewicka D. (2011). Innovation and organisational trust: Study of firms in Poland. Int. J. Innov. Learn..

[57] Sankowska A. (2013). Relationships between organizational trust, knowledge transfer, knowledge creation, and firm’s innovativeness. Learn. Organ..

[58] Curşeu P.L., Schruijer S.G. (2010). Does conflict shatter trust or does trust obliterate conflict? Revisiting the relationships between team diversity, conflict, and trust. Group Dyn. Theory Res. Pract..

[59] Porter L.W., Smith F.J. (1968). The etiology of organizational commitment: A longitudinal study of initial stages of employee-organization relationships. Unpubl. Manuscr..

[60] Sheldon M.E. (1971). Investments and Involvements as Mechanisms Producing Commitment to the Organization. Adm. Sci. Q..

[61] Adebayo O. (2010). Obstetric Nurses’ Perceptions of Manager’s Leadership Style on Job Satisfaction and Organizational Commitment.

[62] Akbolat M., Isik O., Yilmaz A., Akca N. (2015). The Effect of Organizational Justice Perception on Job Satisfaction of Health Employees. Int. J. Recent Adv. Organ. Behav. Decis. Sci..

[63] Avolio B.J., Zhu W., Koh W., Bhatia P. (2004). Transformational leadership and organizational commitment: Mediating role of psychological empowerment and moderating role of structural distance. J. Organ. Behav..

[64] Dunn M.W., Dastoor B., Sims R.L. (2012). Transformational leadership and organizational commitment: A cross-cultural perspective. J. Multidiscip. Res..

[65] Yiing L.H., Ahmad K.Z.B. (2009). The moderating effects of organizational culture on the relationships between leadership behaviour and organizational commitment and between organizational commitment and job satisfaction and performance. Leadersh. Organ. Dev. J..

[66] Rusliza Y., Fawzy E. (2016). Leadership styles and organizational commitment: Literature review. J. Manag. Dev..

[67] Lok P., Crawford J. (2004). The effect of organisational culture and leadership style on job satisfaction and organisational commitment: A cross-national comparison. J. Manag. Dev..

[68] Stum D.L. (1999). Workforce commitment: Strategies for the new work order. Strategy Leadersh..

[69] Nangoli S., Muhumuza B., Tweyongyere M., Nkurunziza G., Namono R., Ngoma M., Nalweyiso G. (2020). Perceived leadership integrity and organisational commitment. JMD.

[70] Kaiser R.B., Hogan R. (2010). How to (and how not to) assess the integrity of managers. Consult. Psychol. J. Pract. Res..

[71] Lo M.C., Min H.W. (2009). Leadership styles and organizational commitment: A test on Malaysia manufacturing industry. Afr. J. Mark. Manag..

[72] Lin W. Innovation Climate, Organizational Commitment and Turnover Intention—Survey on Cultural Enterprises Employees. Proceedings of the 2016 3rd International Conference on Economics and Management (ICEM 2016).

[73] Gagné M., Deci E.L. (2005). Self-determination theory and work motivation. J. Organ. Behav..

[74] Demircioglu M.A., Audretsch D.B. (2017). Conditions for innovation in public sector organizations. Res. Policy.

[75] Holliman S.L. (2012). Exploring the Effects of Empowerment, Innovation, Professionalism, Conflict, and Participation on Teacher Organizational Commitment. Ph.D. Thesis.

[76] Im T., Campbell J.W., Jeong J. (2016). Commitment intensity in public organizations: Performance, innovation, leadership, and PSM. Rev. Public Pers. Adm..

[77] Graen G., Uhl-Bien M. (1995). Relationship-based approach to leadership: Development of leader-member exchange (LMX) theory of leadership over 25 years: Applying a multi-level multi-domain perspective. Leadersh. Q..

[78] Porter L.W., Steers R.M., Mowday R.T., Boulian P.V. (1974). Organizational commitment, job satisfaction, and turnover among psychiatric technicians. J. Appl. Psychol..

[79] Huff L., Kelley L. (2003). Levels of Organizational Trust in Individualist Versus Collectivist Societies: A Seven-Nation Study. Organ. Sci..

[80] De Jong J., Den Hartog D. (2010). Measuring Innovative Work Behaviour. Creat. Innov. Manag..

[81] Brislin R.W. (1980). Cross-cultural research methods. Environment and Culture.

[82] Hair J.F., Page M., Brunsveld N. (2019). Essentials of Business Research Methods.

[83] Hair J.F., Hult G.T.M., Ringle C.M., Sarstedt M. (2022). A Primer on Partial Least Squares Structural Equation Modeling (PLS-SEM).

[84] Hair J.F., Black W.C., Babin B.J., Anderson R.E. (2019). Multivariate Data Analysis.

[85] Hair J.F., Black W.C., Babin B.J., Anderson R.E. (2014). Multivariate data analysis: Pearson new international edition. Essex. Pearson Educ. Ltd..

[86] Ringle C., Steers R., Becker J. SmartPLS. https://www.smartpls.com/.

[87] Lavrakas P.J. (2010). An Evaluation of Methods Used to Assess the Effectiveness of Advertising on the Internet. Interactive Advertising Bureau Research Papers.

[88] Henseler J., Ringle C.M., Sarstedt M. (2015). A new criterion for assessing discriminant validity in variance-based structural equation modeling. J. Acad. Mark. Sci..

[89] Dirks K.T., Skarlicki D.P. (2004). Trust in leaders: Existing research and emerging issues. Trust Distrust Organ. Dilemmas Approaches.

[90] Zhang X., Zhou J. (2014). Empowering leadership, uncertainty avoidance, trust, and employee creativity: Interaction effects and a mediating mechanism. Organ. Behav. Hum. Decis. Process..

[91] Chi J.L., Chi G.C. (2014). Perceived Executive Leader’s Integrity in Terms of Servant and Ethical Leadership on Job Burnout among Christian Healthcare Service Providers. J. Manag. Res..

[92] Park M.-H., Hwang C.-J. (2015). Relationship between servant leadership of nurse managers and positive thinking and organizational commitment of nurses. Korean Comp. Gov. Rev..

[93] Vuong B.N., Tushar H., Hossain S.F.A. (2022). The effect of social support on job performance through organizational commitment and innovative work behavior: Does innovative climate matter?. Asia-Pac. J. Bus. Adm..

[94] Bain P.G., Mann L., Pirola-Merlo A. (2001). The innovation imperative: The relationships between team climate, innovation, and performance in research and development teams. Small Group Res..

[95] Nazir S., Qun W., Hui L., Shafi A. (2018). Influence of social exchange relationships on affective commitment and innovative behavior: Role of perceived organizational support. Sustainability.

[96] Nazir S., Shafi A., Asadullah M.A., Qun W., Khadim S. (2020). Linking paternalistic leadership to follower’s innovative work behavior: The influence of leader–member exchange and employee voice. Eur. J. Innov. Manag..

[97] Park S., Jo S.J. (2017). The impact of proactivity, leader-member exchange, and climate for innovation on innovative behavior in the Korean government sector. Leadersh. Organ. Dev. J..

[98] Isaksen S., Akkermans H.J. (2011). Creative Climate: A Leadership Lever for Innovation. J. Creat. Behav..

